# Validity of ultrasound in predicting acute appendicitis among children, keeping histopathology as gold standard: Methodological issues

**DOI:** 10.1016/j.amsu.2019.05.018

**Published:** 2019-06-14

**Authors:** Roya Karimi, Leila Mounesan, Jamal Rahmani, Siamak Sabour

**Affiliations:** Department of Clinical Epidemiology, School of Health and Safety, Shahid Beheshti University of Medical Sciences, Tehran, Iran; Department of Epidemiology and Biostatistics, Research Centre for Emerging and Reemerging Infectious Diseases, Pasteur Institute of Iran, Tehran, Iran; Department of Community Nutrition, Faculty of Nutrition and Food Technology, National Nutrition and Food Technology Research Institute, Student Research Committee, Shahid Beheshti University of Medical Sciences, Tehran, Iran; Department of Clinical Epidemiology, School of Health and Safety, Shahid Beheshti University of Medical Sciences, Tehran, Iran; Safety Promotions and Injury Prevention Research Centre, Shahid Beheshti University of Medical Sciences, Tehran, Iran

**Keywords:** Acute appendicitis, Ultrasound, Histopathology, Validity, Methodological issues

## Abstract

• It is good to know that sensitivity is an important measure in public health aspects instead of clinical fields. Likewise, PPV and NPV are among the measures which are more appropriate for advice about the validity of a diagnostic test for clinical purposes.
• It is critically important to consider that accurate diagnosis is very important in appendicitis because of the urgent need for surgery in people who are truly positive, likewise, operating surgery for people who are not truly positive will have inconvenient effects.
• Finally, for the prediction of an outcome, we need data from two different cohorts or at least from one cohort divided into two to first to develop a prediction model and subsequently validate it.
• To make our methodological comments brief, it is crucial to know that to determine the validity and predictive ability of ultrasound in predicting acute appendicitis among children, methodological and statistical issues should be correctly taken into account.

We read the article entitled “To determine the validity of ultrasound in predicting acute appendicitis among children, keeping histopathology as the gold standard” by U. Khan et al. published in the Annals of Medicine and Surgery 2019 [1].

The aim of the authors was to determine the accuracy of ultrasound in diagnosis of acute appendicitis in children keeping histopathology as gold standard. They claimed good diagnostic performance for ultrasound by reporting the values of sensitivity (86%), specificity (97%), positive predictive value, PPV (96%) and accuracy (92%).The information obtained from evaluations of the image, operative findings, and pathology of 223 patients with diagnosed appendicitis. They concluded that ultrasound is an accurate model, which causes a significant decrease in negative appendectomies with no increase in the number of CT scans.

However, there are methodological issues which can considerably affect the main message of the study.

First, given the information in the text and Fig 5, the number of the true negatives was not mentioned; therefore, specificity and accuracy of ultrasound cannot be calculated. Surprisingly, they reported specificity and accuracy equal to 97% and 92% respectively. The most appropriate estimates to evaluate validity of a single test such as ultrasound compared to histopathology are sensitivity (Sen), specificity (Spe), positive predictive value (PPV), negative predictive value (NPV), likelihood ratio positive (ranging from 1 to infinity; the higher the LR+, the more accurate the test), likelihood ratio negative (ranging from 0 to 1; the lower the LR-, the more accurate the test), as well as accuracy and odds ratio (ratio of true to false results). According to their results, we will have Sen = 86% and PPV = 96%, but for another parameter we have undefined estimate. It is good to know that sensitivity is an important measure in public health aspects instead of clinical fields. Likewise, PPV and NPV are among the measures which are more appropriate for advice about the validity of a diagnostic test for clinical purposes. Therefore, we suggest applying predictive values, likelihood ratios, odds ratio and diagnostic accuracy to decide about the validity of ultrasound. Moreover, predictive values are dependent on several factors, including disease prevalence, sensitivity and specificity of a test. As the prevalence of the outcome changes, the PPV of the test can easily be affected [[2], [3], [4], [5]]. Accordingly, the author should consider and maybe report the disease prevalence while deriving the results of the study.

Secondly, what is critically important is considering that accurate diagnosis is very important in appendicitis because of the urgent need for surgery in people who are truly positive, likewise, operating surgery for people who are not truly positive will have inconvenient effects. One of the vital facts that are neglected in this article, is the importance of false positive and particularly false negative and considering the consequences of this fact. It must have made authors think of “it might be extremely important when the test diagnoses a person as negative whilst it is positive (false negative)” so that the percent of false negative and specially NPV becomes strikingly critical to consider, calculate correctly and report these cases.

Further, the results that have been expressed on the passage and also a table that is shown in the article are derived from different numbers which have been calculated confusing between appendectomies and gold standard. To clarify this case, I redraw the 2 by 2 table to calculate the true values according to what in the abstract section mentioned.

**Table:** 

		Gold standard (Histopathology)		
		Positive	Negative	Total
Test (Ultrasound)	Positive	TP	FP	192
	Negative	FN	TN	31
	Total	215	8	**223**

As the table illustrates, these pieces of information reported in the article cannot help in calculating the validity measures. This is due to confusion between appendectomies and histopathology as the gold standard. Therefore, the measures which are reported as sensitivity, specificity, and PPV are not correct, consequently, there is a considerable misinterpretation in the result and conclusion of this study that can be significantly important particularly in the clinical field [[2], [3], [4], [5]].

Finally, for the prediction of an outcome, we need data from two different cohorts or at least from one cohort divided into two to first to develop a prediction model and subsequently validate it. Misleading results are generally the main outcome of research that fails to validate its prediction models [6,7]. Therefore, validity estimates such as Sen and PPV, do not guarantee a correct prediction. Because their application is to evaluate the accuracy (validity) of a single test compared to a gold standard considering the value of the rest of validity estimates as well.

In conclusion, the measures which are reported in this article to decide about the validity of the mentioned test are not appropriate, also, interpreting the incorrect results will bring the serious misleading messages as we showed. There are several points which are important to be mentioned not only for authors, but also for other researchers who are working on clinical fields and particularly diagnostic tests. First, recognizing the correct methodology of what is calculating as the measures that are referenced for clinicians and can affect their diagnosis is critically important. Second, the nature of the disease such as its prevalence, severity, infectiousness, etc. should be considered in interpreting the results. Third, in the clinical fields, it is recommended to write a clear sequence and addressing, accurate details, merely for clarifying the correctness of the process of the study.

To make our methodological comments brief, it is crucial to know that to determine the validity and predictive ability of ultrasound in predicting acute appendicitis among children, methodological and statistical issues should be correctly taken into account [[2], [3], [4], [5], [6], [7]].

## Provenance

Not commissioned, externally peer reviewed.

## Source(s) of support

None.

## Disclosure

None declared.

## Conflicts of interest

No potential conflict of interest relevant to this article was reported.

## Ethical approval

N/A.

## Sources of funding

No.

## Author contribution

Roya Karimi, Leila Mounesan and Jamal Rahmani discussed the concept of the issue and wrote the first draft of the paper. Siamak Sabour revised and wrote the final version of the paper.

## Research registry number

N/A.

## Guarantor

Siamak Sabour.
